# Magnetostrictive Bioinspired Whisker Sensor Based on Galfenol Composite Cantilever Beam Realizing Bidirectional Tactile Perception

**DOI:** 10.1155/2018/4250541

**Published:** 2018-07-24

**Authors:** Ran Zhao, Qan-guo Lu, Qinghua Cao

**Affiliations:** ^1^Jiangxi Province Key Laboratory of Precision Drive & Control, Nanchang Institute of Technology, Nanchang, China; ^2^School of Electrical Engineering, Hebei University of Technology, Tianjin, China

## Abstract

A magnetostrictive bioinspired whisker sensor based on a galfenol/beryllium-bronze/galfenol composite cantilever beam was developed in this work. According to the new design concept, the proposed whisker can output positive and negative voltages under different bending directions. Besides, the proposed whisker sensor can realize the bidirectional distance and microforce perception. Using the classical beam theory, a theoretical model was used to predict the output performance of the whisker. An experimental system was established to test the whisker's output performance. In the experiment, the designed whisker, compared with a traditional unimorph whisker, displayed an output voltage range of −240 to 240 mV. The parameters were as follows: the distance was 0–22 mm, with the microforce sensing range of 9.8–2744 mN, the average distance was 10.90 mm/mV, and the force sensitivity was 11.4 mN/mV. At last, obstacle perception was applied. The experiment showed that the proposed whisker sensor can realize the bidirection tactile perception in one-dimensional space. The work expands the function of the magnetostrictive bioinspired whisker, acquiring the multi-information for single-sensor system.

## 1. Introduction

Recently, with the development of robot technology, the bioinspired whisker has attracted the attention of researchers. As a “nonvisual” perception method, it is installed on bionic robots and automatic vehicles, to realize tactile perception, hydrodynamic measurement, and shape sensing [1–5]. Due to the excellent environmental adaptability, the bioinspired whisker, by providing an artificial “tactile,” can collect more ambient information to make up for the lack of machine vision.

Until now, the reported bioinspired whiskers include capacitive whisker, piezoelectric whisker, elastic whisker, and magnetostrictive whisker [6–11]. Wherein, magnetostrictive whisker shows the advantages of working under static or low-frequency conditions [12, 13], comparing to piezoelectric and capacitive whiskers.

However, for traditional magnetostrictive whisker, its structure and principle (the position of magnetism sensor and the measuring method) determine that in the following condition—when bending to different directions, the output signals are asymmetrical. Thus, it is insensitive to the directions of stress and vibration, which limits its application.

Due to this, we developed a magnetostrictive whisker sensor, which is basing on a composite cantilever beam with sandwich structure. The operation principle was analyzed, compared to that of traditional magnetostrictive whisker. We used two Hall sensors to generate a differential output, thus realizing the detection of bidirectional bending. The experimental system was built to test the proposed whisker.

## 2. Design

### 2.1. Structure

**Figure 1:**
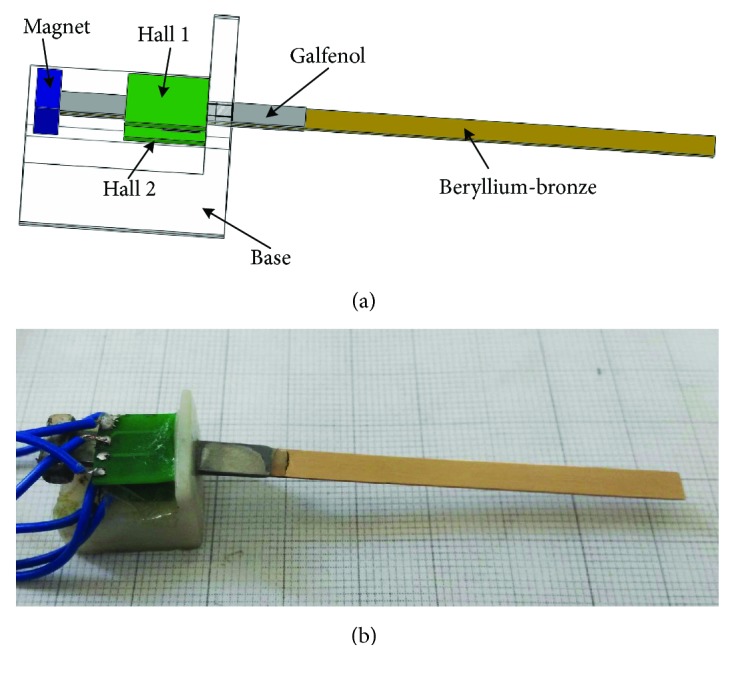
Magnetostrictive whisker sensor: (a) 3-D model and (b) photography.

Figure 1 gives the structure of the whisker, consisting of a galfenol/beryllium-bronze/galfenol composite cantilever beam, a magnet, two Hall sensors, and a base. The magnet uses a bias magnetic field to magnetize two galfenol sheets. When a stress is applied, the flux linkage change induced by the bending beam is measured by the two Halls. The composite beam is fixed by the base with an optimal distance between the magnet and the clamped point [12], enabling the Hall to detect the maximum change flux.

The composite cantilever beam was fabricated by a long beryllium-bronze beam as substrate, with two short galfenol beams bonding on it. The beryllium-bronze substrate provides excellent elasticity, making the composite beam bear larger stress and higher vibration frequency than the single beam. Tables 1 and 2 show the physical and geometry parameters of the proposed whisker, respectively.

### 2.2. Principle

**Figure 2:**
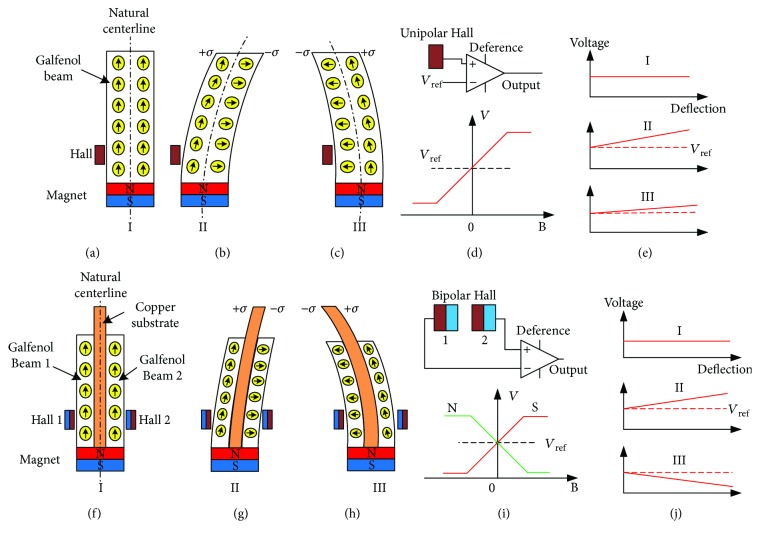
Principle of the traditional and proposed magnetostrictive bioinspired whisker: structure, operation mode, measurement method, and output signal.

Figure 2 shows the working principle of traditional, newly designed magnetostrictive whiskers. It can find the way to realize the bidirection detection.  Figure 2(a) shows a conventional structure of magnetostrictive whisker developed by Flatua; there is only one Hall sensor used to detect the linkage flux. Thus, we can know how the magnetic domains rotate with the application of a stress (observed by Raghunat et al. by using Kerr microscope [14]).

Figures 2(b) and 2(c) (named “mode I” and “mode II”) show that these rotations are different for magnetic domains distributed on both sides of the natural center line. Using a unipolar Hall to detect the linkage flux (see Figure 2(d)), the output signal is asymmetric. Furthermore, the output signal of mode I is larger than that of mode II (see Figure 2(e)). It is the reason why the traditional magnetostrictive whisker cannot identify the directions of loading stress.

Defining the output of Hall as *U*_h_ and the reference voltage (when magnetic flux is zero) as *U*_ref_ (see Figure 2(a)), we can obtain the final output signal with a different operation.

(1) $$\begin{array}{c} U_{uni}=U_{h}-U_{ref}. \end{array}$$

For the given whisker, a composite beam is used instead of the original single beam.  Figure 2(f) shows the natural center line is in the middle of the beryllium-bronze beam. It makes the rotation of the bending magnetic domains consistent in each galfenol beam.

Moreover, the changes of linkage flux for each galfenol beam are measured by different Hall sensors (see Figures 2(g) and 2(h)). Two bipolar Halls are used to realize the magnetic field detection.  Figure 2(i) shows that bipolar Hall has two different outputs of north pole and south pole (abbreviated *N* and *S*), which are used to implement differential output. Supposing *N* output of Hall 1 as *U*_h1_ and *S* output of Hall 2 as *U*_h2_, the final output signal is

(2) $$\begin{array}{c} U=U_{h1}-U_{h2}-U_{ref}. \end{array}$$

The new measuring circuit is used to obtain a signal with positive and negative symmetry (see Figure 2(j)), which makes the proposed whisker sensor identify the force directions.

## 3. Model

**Figure 3:**
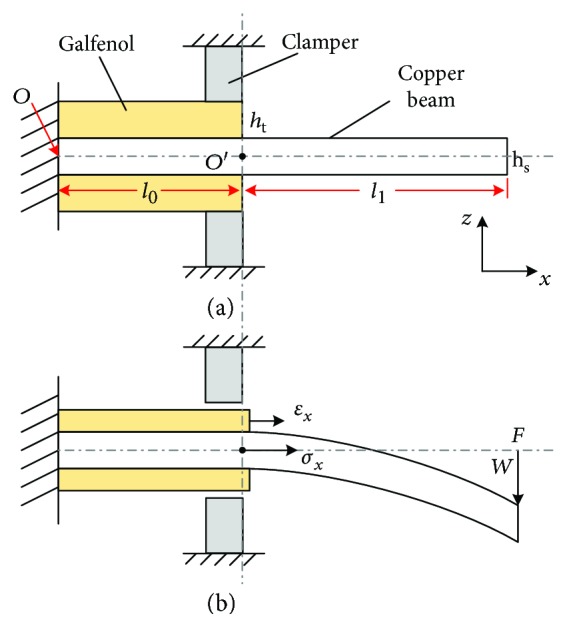
Bending model of the composite cantilever beam.

Figure 3 shows a simplified bending model of the galfenol/beryllium-bronze/galfenol composite beam. Figure 3(a) shows that the proposed model is different from the conventional beam model—there is a clamper to limit the starting location of the bending beam (with the origin of coordinate changing from O to O′).

Based on the 2-dimensional coordinate of the beam (shown in Figure 3(a)), *l* is the total length of the substrate beam; *h*_s_ is the thickness; *l*_0_ is the length of galfenol beam; and *h*_t_ is the thickness: *l* = *l*_0_ + *l*_1_.

Figure 3(b) shows how the beam bends when a force is applied at the beam's free end. It is assumed that *F* is the loading force and *w* is the deflection. At the coordinate point *O*′, *σ*_*x*_ is the tensile stress along the *x-*direction of the beam and *ε*_*x*_ is the strain of galfenol beam under the tensile stress.

According to the Euler-Bernoulli beam theory, the equivalent tensile stress of the bending beam at O′ can be expressed as [15]

(3) $$\begin{array}{c} \sigma_{x}={zE}_{s}I\frac{d^{2}w}{{dx}^{2}}=z\frac{F\left(L_{1}-x\right)}{I}, \end{array}$$

where *z* is the distance between the position to the natural center line of the composite beam along *z*-direction; *x* is the position at *x*-axis; *E*_s_ is the Young's modulus of the substrate beam; and *I* is the second moment of area of the beam's cross section.

The relation of loading force and deflection is expressed as

(4) $$\begin{array}{c} w=\frac{F}{3E_{s}I}L_{1}^{3}. \end{array}$$

Figure 2(a) shows that there is a composite cantilever beam with sandwich structure from *O* to *O*′. The effective Young's modulus of the composite beam can be obtained by

(5) $$\begin{array}{c} E_{e}=\frac{2E_{t}h_{t}+E_{s}h_{s}}{2h_{t}+h_{s}}, \end{array}$$

where *E*_t_ is the Young's modulus of galfenol beam.

The elastic relation can be expressed by Hook's law, *σ* = *E* · *ε*, and there is

(6) $$\begin{array}{c} \varepsilon_{x}=\frac{\sigma_{x}}{E_{e}}=\frac{2h_{t}+h_{s}}{2E_{t}h_{t}+E_{s}h_{s}}\sigma_{x}. \end{array}$$

Using the piezomagnetic equation, Downey and Flatau and Datta and Flatau derived the expression of magnetic flux [16, 17], that is,

(7) $$\begin{array}{c} B=d_{33}^{*}E_{t}\varepsilon_{x}-d_{33}d_{33}^{*}E_{t}H_{0}+\mu_{0}\mu_{r}H_{0}, \end{array}$$

where *d*_33_ is the piezomagnetic coefficient; *d*_33_^∗^ is the inverse piezomagnetic coefficient; *H*_0_ is the bias magnetic field; *μ*_0_ is the permeability of vacuum; and *μ*_r_ is the relative permeability of galfenol.

Substituting (7) into (2), we can obtain

(8) $$\begin{array}{c} U=2s_{H}B=2s_{H}d_{33}^{*}E_{t}\varepsilon_{x}, \end{array}$$

where *s*_H_ is the sensitivity of Hall sensor.

According to (3), (4), (5), and (6), we derive the expressions of output voltage in different loading conditions as follows:

(9) $$\begin{array}{c} U_{\sigma }=2s_{H}d_{33}^{*}\frac{E_{t}\left(2h_{t}+h_{s}\right)}{2E_{t}h_{t}+E_{s}h_{s}}\frac{{zL}_{1}}{I}F, \end{array}$$

(10) $$\begin{array}{c} U_{d}=2s_{H}d_{33}^{*}\frac{3E_{t}E_{s}\left(2h_{t}+h_{s}\right)}{2E_{t}h_{t}+E_{s}h_{s}}\frac{z}{L_{1}^{3}}w. \end{array}$$

## 4. Experiment

The work studied the static performances of the proposed sensor. An experimental system was established to test the relations between deflection-voltage and force-voltage, thus investigating the tactile perception of the whisker sensor. Additionally, a dynamic contact experiment was performed to investigate its obstacle identification.

**Figure 4:**
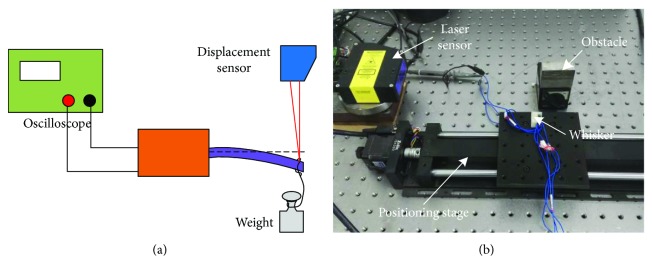
Experimental system for (a) deflection and force test and (b) contact test.

Figure 4 shows the experimental system. The deflection was measured when the beam bends, with the output signal recorded by an oscilloscope (see Figure 4(a)). The largest deflection was limited to 22 mm, thus preventing the beam from damage. With a traditional load-bearing experiment method (hanging standard weights), the microforce sensing performance of the whisker sensor was studied, and the weights changed from 1 to 280 g. The sensor's distance (deflection) and force sensitivity were obtained by the two experiments.

Figure 4(b) shows the experimental system of dynamic contact; the whisker was fixed on a motion stage, with a simulated obstacle placed on its motion path. The deflection was measured by a laser displacement sensor (Lts-250). Besides, the voltage was recorded by an oscilloscope. This experiment shows how the sensor works when a dynamic contact occurs. It can simulate not only the active exploration for the unknown obstacle (such as a whisker system installed on a robot rat [2]) but also the passive perception for external contact force changes. Both refers to a bionic tactile.

## 5. Results and Discussion

**Figure 5:**
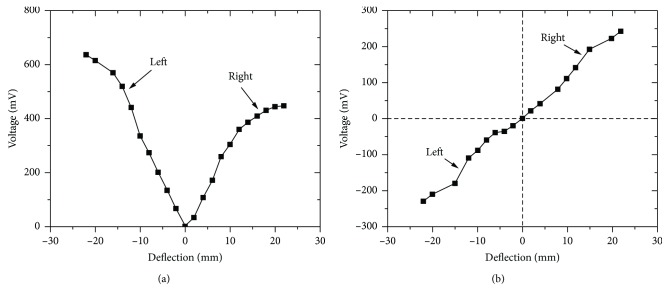
Deflection-voltage curve of (a) traditional and (b) newly designed magnetostrictive whisker sensor.

Firstly, we compared the performance of the magnetostrictive whisker with traditional structure and newly designed structure. In Figure 5, for the traditional one (see Figure 5(a)), the maximum output voltage is 640 mV at a deflection of 22 mm, when the beam bends to the left. When it bends to another direction, the output voltage is positive, but smaller than the former. From Section 2, it can be found that the asymmetry of the curve is caused by the inhomogeneity of magnetic domain rotation and the position of the Hall sensor.

The deflection-voltage curve shows a nearly linear relationship and changes from negative to positive with the changed bending direction (see Figure 5(b)). The maximum output voltage is 240 mV at a deflection of 22 mm. The average deflection sensitivity of the proposed whisker sensor is 10.9 mV/mm.

**Figure 6:**
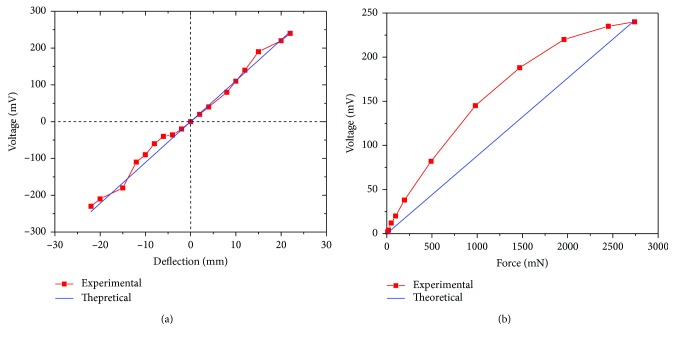
Comparison of the experimental and theoretical sensing performance for (a) distance and (b) force sensing.

In Figure 6, we compared the experimental and theoretical result for distance and microforce sensing. As shown in Figure 6(a), the theoretical calculation is closed to the experimental result; however, there is an error between them in Figure 6(b). As in the distance sensing experiment, the whisker sensor is working at its linear region. For force sensing test, the whisker sensor reaches its saturated region. Equations (9) and (10) provide a linear description. In fact, in these two equations, the inverse piezomagnetic coefficient is not a constant, which depends on the value of the magnetic field and stress. Therefore, the prediction will be more accurate if we replace the constant with a function of *d*_33_^∗^.

Figure 6 shows the microforce sensing performance of the whisker sensor. The proposed whisker is tested when the load changes from 9.8 to 2744 mN. When the applied force is 2744 mN, the output voltage is 240 mV. The average force sensitivity is 11.42 mN/mV, and the proposed sensor has a resolution of 9.8 mN/2 mV.

**Figure 7:**
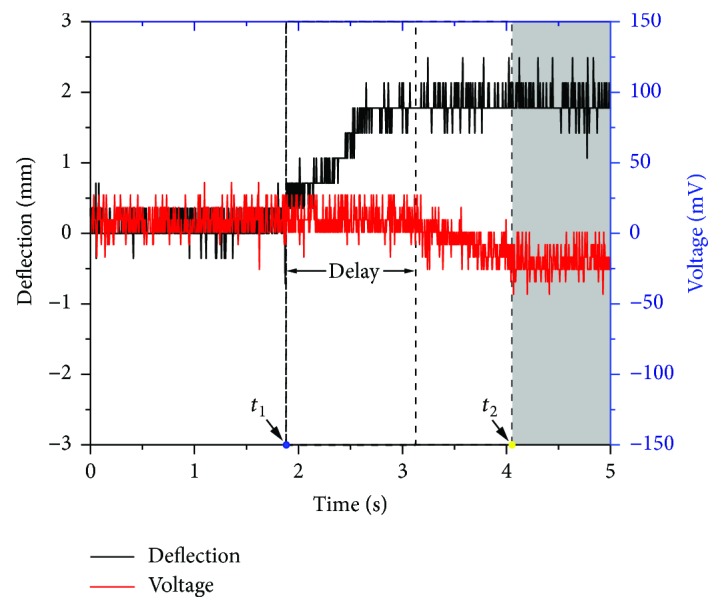
Obstacle perception process of the proposed whisker sensor.

Figure 7 shows the obstacle perception of the proposed whisker sensor. The whisker sensor is fixed on the motion stage and moves at a constant speed. The dynamic contact process can be divided into three stages: (a) at stage 1 (0~*t*_1_), there was no contact between the sensor and obstacle; (b) at stage 2 (*t*_1_*~t*_2_), the beam contacted the obstacle and begun to bend; and (c) at stage 3 (after *t*_2_), when the control unit has detected the raising voltage, the moving process was stopped immediately. In this test, we focused on the response time of the whisker sensor in the contact process.

There is a delay time (*t*_d_) between the deflection and sensing voltage. The delay time can be calculated by *w*_min_/*v*, where *w*_min_ is the minimum deflection of the cantilever beam measured by Hall sensor and *v* is the relative vocasity between the whisker sensor and obstacle. After that, the sensing voltage increases, and an addition time (*t*_p_) is needed for the measuring circuit to test the voltage changing from zero (which is usually very short and can be ignored). As a result, we can define the total perception time as

(11) $$\begin{array}{c} t_{tot}=t_{d}+t_{p}\approx \frac{w_{\min}}{v}. \end{array}$$

Equation (11) indicates that the total perception time *t*_tot_ is mainly due to the relative speed *v*. This experiment indicates that the proposed whisker can detect the obstacles.

## 6. Conclusions

In this work, a bioinspired magnetostrictive whisker was proposed. Based on the new structure composite cantilever beam and differential measurement method, the proposed whisker realized the perception of bidirectional tactile. To testify the operation principle, an experimental system was established. Furthermore, the static performances of the proposed whisker sensor were tested. The designed whisker has properties as follows:

- The distance measurement range is 2–22 mm, with sensitivity of 10.9 mV/mm.
- The microforce sensing range is 9.8–2744 mN, with force sensitivity of 11.4 mN/mV and resolution of 9.8 mN (at 2 mV).
- It has bidirectional perception.

Our research expands the function of magnetostrictive bioinspired whisker. Applied to dynamic measurement, the given sensor can detect more information than existing whisker sensors. The “multi-information sensing technique” based on bioinspired magnetostrictive whisker will be deeply studied.

## Figures and Tables

**Table 1:** Physical parameters of the whisker.

Material	Parameter	Value (unit)
Galfenol	Magnetostrictive coefficient	220 ppm
	Young's modulus	70 GPa
	Poisson's ratio	0.35
Beryllium-bronze	Young's modulus	128 GPa
	Poisson's ratio	0.42
Bias magnet	Bias magnetic field	190 mT
Hall	Measurable range	±150 Gauss

**Table 2:** Geometric parameters of the whisker.

Component	Material/type	Dimensions (mm)
Composite beam	Galfenol	30 × 4 × 0.2
	Beryllium-bronze	80 × 4 × 0.3
Bias magnet	NdFeB	8 × 4 × 3
Base	PVC	20 × 25 × 20
Hall	WH202	—

## Data Availability

The data used to support the findings of this study are included within the article.
